# E3 Ubiquitin Ligase RLIM Negatively Regulates c-Myc Transcriptional Activity and Restrains Cell Proliferation

**DOI:** 10.1371/journal.pone.0164086

**Published:** 2016-09-29

**Authors:** Rui Gao, Lan Wang, Hao Cai, Jingjing Zhu, Long Yu

**Affiliations:** 1 State Key Laboratory of Genetic Engineering, Institute of Genetics, School of Life Sciences, Fudan University, Shanghai, P.R. China; 2 Shanghai Center for Systems Biomedicine, Key Laboratory of Systems Biomedicine Ministry of Education, Shanghai Jiao Tong University, Shanghai, P.R. China; 3 Key Laboratory of Nutrition and Metabolism, Institute for Nutritional Sciences, Shanghai Institutes for Biological Sciences, Chinese Academy of Sciences, Shanghai, P.R. China; University of Miami School of Medicine, UNITED STATES

## Abstract

RNF12/RLIM is a RING domain-containing E3 ubiquitin ligase whose function has only begun to be elucidated recently. Although RLIM was reported to play important roles in some biological processes such as imprinted X-chromosome inactivation and regulation of TGF-β pathway etc., other functions of RLIM are largely unknown. Here, we identified RLIM as a novel E3 ubiquitin ligase for c-Myc, one of the most frequently deregulated oncoproteins in human cancers. RLIM associates with c-Myc *in vivo* and *in vitro* independently of the E3 ligase activity of RLIM. Moreover, RLIM promotes the polyubiquitination of c-Myc protein independently of Ser62 and Thr58 phosphorylation of c-Myc. However, RLIM-mediated ubiquitination does not affect c-Myc stability. Instead, RLIM inhibits the transcriptional activity of c-Myc through which RLIM restrains cell proliferation. Our results suggest that RLIM may function as a tumor suppressor by controlling the activity of c-Myc oncoprotein.

## Introduction

RLIM is a RING domain-containing E3 ubiquitin ligase first reported to play an important role in the chicken limb development by controlling CLIM abundance [1]. More recent research revealed its new functions. In Xenopus, Rlim maintains proper stoichiometry of Xlim-1/Ldb1 and confers proper function of the Spemann organizer [2]. It modulates telomere length homeostasis through proteolysis of TRF1 [3]. RLIM and CLIM interact with estrogen receptor α (ERα) and regulate its target gene expression [4]. RLIM was also identified as a component of the TGF-β superfamily signaling pathways [5, 6]. It controls embryonic stem cell fate and morphogenesis in Zebrafish embryos by targeting the negative regulator Smad7 for proteasomal degradation [6]. Conditional knockout mouse model revealed that paternal Rnf12/RLIM is a critical survival factor for milk-producing alveolar cells [7]. The most exciting finding was that RLIM initiates imprinted X-chromosome inactivation (iXCI) by targeting REX1 for degradation [8, 9]. However, it is dispensable for random form of XCI (rXCI) in mouse embryonic epiblast cells around implantation stage [10]. Our lab recently found that RLIM promotes cell migration *in vitro* by regulation of TGF-β pathway [11]. Moreover, we identified an interplay between p53 and RLIM: p53 represses the transcription of *Rnf12* through interfering with the transcriptional activity of Sp1 [12]. On the other hand, RLIM enhances p53 stability and activity by targeting MDM2 for degradation [13]. However, other functions of RLIM are not well understood. Especially, the substrates for RLIM as an E3 ubiquitin ligase are poorly defined.

c-Myc is a multifunctional transcription factor that plays fundamental roles in proliferation, apoptosis, tumorigenesis, and stem cell pluripotency [14]. *MYC* is documented to be involved broadly in many cancers, in which its expression is estimated to be elevated or deregulated in up to 70% of human cancers [15]. Thus it is not surprising that Myc abundance is tightly controlled. Myc protein is rapidly degraded following its synthesis (half-life of 20 min in non-transformed cells) [16]. One of the most prominent mechanisms to control proper Myc level is degradation by the ubiquitin-proteasome system [17]. Many E3 ligases have been reported to control Myc stability and activity. FBW7, SKP2, HECTH9, TRUSS, PIRH2, CHIP and FBXO32 mediate degradation of Myc, while β-TrCP and FBXO28 promote Myc stabilization [18–30]. Functionally, SKP2, HECTH9, FBXO28, β-TrCP promote Myc transcriptional activity, while others inhibit Myc function [3, 6–17]. Phosphorylation also regulates c-Myc stability. The best characterized interplay between phosphorylation and ubiquitination of c-Myc is phosphorylation of Ser62 and Thr58 and ubiquitination by FBW7 during cell cycle progression [31, 32]. When cells are stimulated to enter cell cycle, phosphorylation at Ser62 by ERK stabilizes c-Myc and enhances its transcriptional activity. Later in G1 phase, Gsk-3β phosphorylates c-Myc on Thr58, which is dependent on prior phosphorylation of Ser62 and promotes polyubiquitination and degradation of c-Myc by FBW7 [32, 33].

In this study, we identified c-Myc as a novel binding partner and substrate for RLIM. RLIM catalyzes non-degradation-associated polyubiquitination of c-Myc independently of c-Myc phosphorylation on Ser62 and Thr58. RLIM-mediated ubiquitination has no effect on c-Myc stability. Instead, it inhibits c-Myc transcriptional activity. Moreover, RLIM restricts cell growth by regulation of c-Myc. Our findings reveal a tumor suppressor role for RLIM which could potentially be exploited for cancer treatment.

## Materials and Methods

### Plasmids and antibodies

RLIM and c-Myc expression plasmids were constructed by cloning human *RLIM* (NM_016120) and *c-MYC* (NM_002467) ORF into pCMV-HA (Clontech) and pCMV-myc (Clontech) vectors respectively. RLIM^C596A^ and c-Myc^T58A/S62A^ expression plasmids were constructed by target point mutagenesis (Strategene). Human ubiquitin ORF were cloned into pCMV-flag (Clontech) and pcDNA3.1-his (ThermoScientific) vectors respectively. For bacterial expression, *RLIM* and *c-MYC* ORF were cloned into pET28a (6×His) (Clontech) and pGEX-4T-2 (GE Healthcare Life Sciences) vectors respectively.

The antibodies used were anti-RLIM (M01, Abnova, 1:1000), anti-c-Myc (9E10, Santa Cruz, 1:200), anti-HA (Roche, 1:1000), anti-myc (Clontech, 1:1000), anti-Flag (M2, Sigma, 1:1000), anti-actin (Sigma, 1:10000) and anti-GFP (Santa Cruz, 1:1000).

### Cell lines

Human osteosarcoma cell line U2OS (HTB-96), human embryonic kidney cell line 293T (CRL-3216) and human lung cancer cell line H1299 (CRL-5803) were obtained from the American Type Culture Collection (ATCC, Manassas, VA).

### Cell culture

U2OS and 293T cells were cultured in Dulbecco’s modified Eagle’s medium (DMEM) (ThermoFisher Scientific) supplemented with 10% fetal bovine serum (FBS, ThermoFisher Scientific) and 1% penicillin/streptomycin (Sigma) at 37°C in a humidified incubator with 5% CO2 (v/v). H1299 cells were cultured in RPMI-1640 medium (ThermoFisher Scientific) supplemented with 10% fetal bovine serum (FBS) and 1% penicillin/streptomycin (Sigma) at 37°C in a humidified incubator with 5% CO2 (v/v).

### Plasmid and siRNA transfection

The cells were seeded one day before transfection. Lipofectamine 2000 reagent (ThermoFisher Scientific) was used for both siRNA and plasmid transfection when the cells reach to about 80–90% confluency for plasmid transfection and 50–70% confluency for siRNA transfection. Medium was changed after 4–6 hours to complete medium with FBS and P/S. Cells were ready for use after 48 hours incubation. The target sequences for siRNAs were as follows. Scr1, 5-CAT GTC ATG TGT CAC ATC T-3; Scr2, 5-CAT GTC ATG TGT CAC ATC T-3; siRLIM1, 5-GTT CCA GTT CCA GTC CTA G-3; siRLIM2, 5-GGC TTA TGA GAG ATA ACA A-3; siRLIM3, 5-CTG CAT CGA TCG CTG GTT A-3; siRLIM4, 5-GCA ATT CAG ACC ATG TTA A-3; si-c-Myc1, 5-CAT CAT CAT CCA GGA CTG TAT-3; si-c-Myc2, 5-CGA GCT AAA ACG GAG CTT T-3.

### Co-immunoprecipitation and Immunoblotting

Cells were lysed with 1 × cell lysis buffer (Cell Signaling Technology) and rotated at 4°C for 30 min. Cell debris was removed by centrifugation and the soluble fraction was collected and precleared with Protein A/G Agarose beads for 2 hours at 4°C. The precleared cell lysate was incubated with indicated antibodies overnight followed by incubation with Protein A/G beads for at least 2 hours at 4°C. Immunoprecipitates were then washed 6 times with cell lysis buffer and boiled in 1 × SDS loading buffer. Samples were resolved on SDS-PAGE and transferred to PVDF membranes and immunoblotting was carried out with antibodies as indicated.

### GST pull down assay

The human *RLIM* gene was subcloned into pET28a (6×His)expression plasmid and the human *c-MYC* gene was subcloned into pGEX-4T-2 expression plasmid with GST tag (pGEX-4T-2 empty vector as a negative control). Recombinant proteins were induced to be expressed in *Escherichia coli* strain BL21 (DE3) by 0.1mM IPTG for 1–2 hours at 28°C (GST and His-tagged RLIM) or 25°C (GST tagged c-Myc). The bacteria were lysed by lysis buffer (1mM DTT, 1mM PMSF, 1% Triton in 1x PBS). GST or GST-c-Myc protein-containing cell lysate were incubated with equal amount of GST-Sepharose (GE Healthcare Life Sciences) for 4 hours at 4°C. After washing the beads 6 times with GST-pull down buffer (50 mM Tris-HCl [pH 7.5], 150 mM NaCl, 1 mM DTT, and 0.01% Triton X-100), the supernatant was removed by centrifugation. Then equal amount of 6 × His-RLIM protein-containing E. coli cell lysate was added into the beads and incubate at 4°C for at least 1 hour. After 6 times washing with GST-pull down buffer, proteins bound to the beads were analyzed by SDS-PAGE followed by immunoblotting with anti-His antibody.

### Construction of stable cell lines

U2OS cells were transfected with pcDNA3.1 or pcDNA3.1-HA-RLIM plasmid. Stably transfected cells were selected by G418 resistance (800μg/mL) for 2 weeks. Mixed clones were used for experiments.

### Cell Proliferation Assay

The cells were plated in 6 replicates at a density of 1500–3000 cells per well in a 96 well plate. Cell proliferation was monitored by the Cell Counting Kit-8 (CCK-8) as instructed. The amount of formazan produced which was directly proportional to the number of living cells was measured by absorbance at 490 nm in a microplate reader. The measurement was taken every 24 hours for 3 or 4 consecutive days.

### Dual-Luciferase Reporter Assay

293T cells were seeded in 4 replicates at a density of 6–8 × 10^4^ cells per well in 24 well plates. 24 hours later, cells were transfected with myc-c-Myc plasmid and/or HA-RLIM plasmid together with an E-box reporter plasmid as well as a control reporter vector pRL-TK (Promega). After 30 hours incubation, the cells were harvested and lysed with 100ul 1 × PLB (passive lysis buffer, Promega) for 15min at room temperature with gentle shaking. Luciferase assays were performed by Luminometer LB9507. Firefly and Renilla luciferase activity was performed sequentially with a 10 second interval. Luciferase activity was analyzed based on the ratio of Firefly/Renilla (co-transfecting pRL-TK vector as an internal control to normalize cell number and transfection efficiency) and the 4 replicates were averaged.

### Quantitative PCR

RNA was isolated with the RNeasy kit (QIAGEN). RNA was reversed transcribed using SuperScript III First-Strand Synthesis System (ThermoFisher Scientific) and random hexamers. cDNA was then subjected to quantitative PCR using SYBR Green PCR Master Mix (ThermoFisher Scientific). Primer sequences were as follows. *E2F2*, forward 5-ACA AGG CCA ACA AGA GGC TG-3, reverse 5-TCA GTC CTG TCG GGC ACT TC-3; *Nucleolin*, forward 5-ACT GAC CGG GAA ACT GGG TC-3, reverse 5-TGG CCC AGT CCA AGG TAA CT-3; *ActB*, forward 5-TCC CTG GAG AAG AGC TAC GA-3, reverse 5-AGC ACT GTG TTG GCG TAC AG-3.

### Statistical Analysis

Experiments were performed at least three times and representative results were shown. Statistical analysis was performed with a two-tailed unpaired t test or one-way ANOVA. p< 0.05 was considered statistically significant.

## Results

### RLIM interacts with c-Myc in cells and *in vitro*

**Fig 1:**
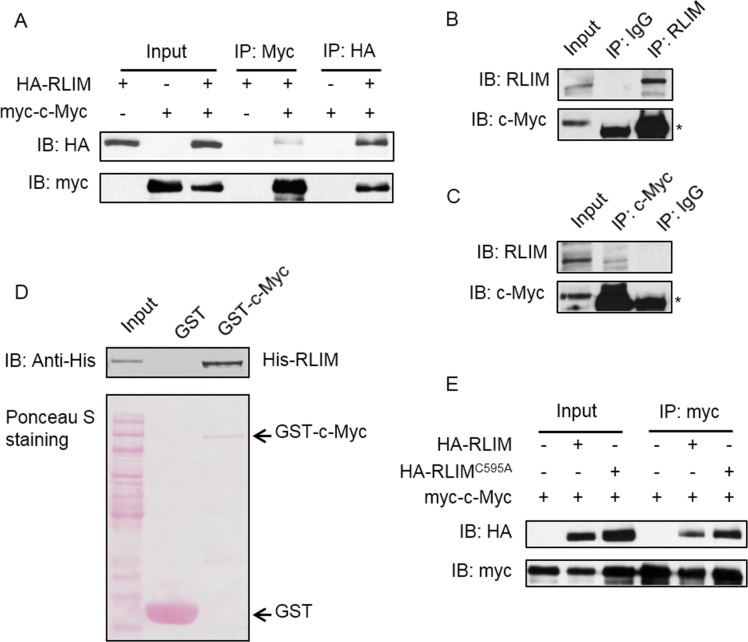
RLIM interacts with c-Myc. (A) 293T cells were transfected with HA-RLIM and myc-c-Myc expression vectors and immunoprecipitation was carried out with anti-myc or anti-HA antibodies as indicated. Immunoprecipitates were subjected to WB with anti-HA and anti-myc antibodies. (B) and (C) 293T cell lysate was subjected to immunoprecipitation with control IgG antibody and anti-RLIM (B) or anti-c-Myc (C) antibodies. Immunoprecipitates were subjected to WB with anti-c-Myc and anti-RLIM antibodies. * indicates the heavy chain of IgG. (D) Purified bacterial-expressed GST or GST-c-Myc proteins were incubated with bacterial-expressed His-RLIM protein. Interaction between RLIM and c-Myc were detected by GST pull-down and subsequent WB with anti-His antibody. (E) 293T cells were transfected with WT or catalytic dead HA-RLIM together with myc-c-Myc. Cells were subjected to immunoprecipitation with anti-myc antibody followed by WB with anti-HA and anti-myc antibodies.

To study the interaction between RLIM and c-Myc, we transfected 293T cell with HA-RLIM and myc-c-Myc expression plasmids alone or together and performed a set of reciprocal co-IP-IB assays with myc and HA antibodies respectively. HA-RLIM and myc-c-Myc were specifically coimmunoprecipitated by myc and HA antibodies respectively only in cells with co-expression of both, but not in cells that express either one alone (Fig 1A). Consistent with these results, endogenous RLIM also bound to endogenous c-Myc (Fig 1B and 1C). These results indicate RLIM indeed associates with c-Myc. To access whether the two proteins interact with each other directly, we performed GST pull-down assay using bacteria-expressed His-RLIM and GST-c-Myc proteins. GST-c-Myc but not GST alone specifically pulled down His-RLIM protein (Fig 1D), which demonstrated the direct association of the two proteins. In addition, we constructed a catalytic dead RLIM expression plasmid by mutating an essential amino acid in the catalytic domain (C596A), and performed the co-IP assay. We found that RLIM^C596A^ still interacted with c-Myc, suggesting that the E3 ligase activity of RLIM is dispensable for its interaction with c-Myc (Fig 1E). Together, these results demonstrate that RLIM is a bona fide binding partner of c-Myc.

### RLIM promotes c-Myc ubiquitination

**Fig 2:**
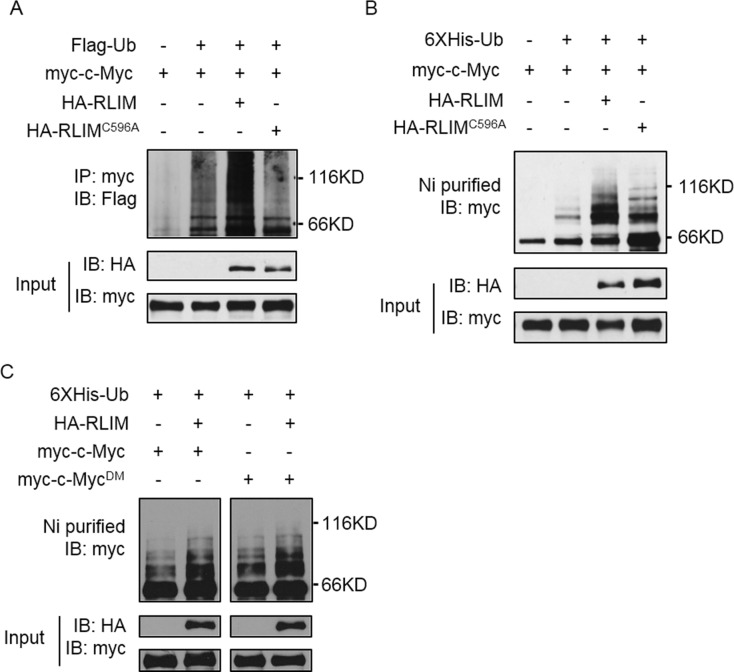
RLIM promotes c-Myc ubiquitination. (A) 293T cells were transfected with Flag-Ub, myc-c-Myc, HA-RLIM and HA-RLIM^C596A^ in combinations as indicated. Ectopically expressed c-Myc was immunoprecipitated by anti-myc antibody followed by WB with anti-Flag antibody. (B) 293T cells were transfected as in (A) except using 6 × His-Ub instead of Flag-Ub. Ubiquitinated proteins were precipitated with nickel (Ni)-NTA beads and subjected to WB with anti-myc antibody. (C) 293T cells were transfected with His-Ub, HA-RLIM, myc-c-Myc and myc-c-Myc^DM^ (S62A and T58A) as indicated. Ubiquitinated proteins were precipitated with nickel (Ni)-NTA beads and subjected to WB with anti-myc antibody.

Since RLIM is an E3 ligase, we hypothesized that RLIM might serve to ubiquitinate c-Myc *in vivo*. We transfected 293T cells with Flag-ubiquitin (Ub), myc-c-Myc, HA-RLIM and HA-RLIM^C596A^ plasmids in different combinations, then immunoprecipitated myc-c-Myc and examined its ubiquitination status. We found that ubiquitination of c-Myc was significantly increased only by co-expression of WT RLIM but not catalytic-dead RLIM (C596A) (Fig 2A), suggesting that RLIM promotes ubiquitination of c-Myc. To further confirm this, we transfected 293T cells as above except using 6 × His-Ub construct instead of Flag-Ub. We affinity-purified His-tagged proteins under denaturing conditions and blotted with myc antibody. We found there was a significant increase of slow-moving bands only when WT RLIM was co-expressed (Fig 2B). There was some increase of slow-moving bands in RLIM^C596A^ co-expression group (Fig 2B), suggesting the E3 ligase activity of RLIM was not completely abolished by the point mutation. It was reported that phosphorylation of c-Myc on Ser62 and Thr58 regulates its ubiquitination by FBW7. This prompted us to examine whether phosphorylation of c-Myc on Ser62 and Thr58 also affects its ubiquitination by RLIM. We constructed a double point mutation of c-Myc (S62A and T58A) to abolish its phosphorylation on these two sites. Co-expression of WT or double mutant (DM) c-Myc (c-Myc^DM^) with RLIM followed by ubiquitination assay showed that both WT c-Myc and c-Myc^DM^ were ubiquitinated to similar extent by RLIM (Fig 2C), indicating that the ubiquitination of c-Myc by RLIM was not dependent on Ser62 and Thr58 phosphorylation. Taken together, these findings suggest that RLIM is a new E3 ligase for c-Myc.

### RLIM-mediated ubiquitination of c-Myc is not degradative

**Fig 3:**
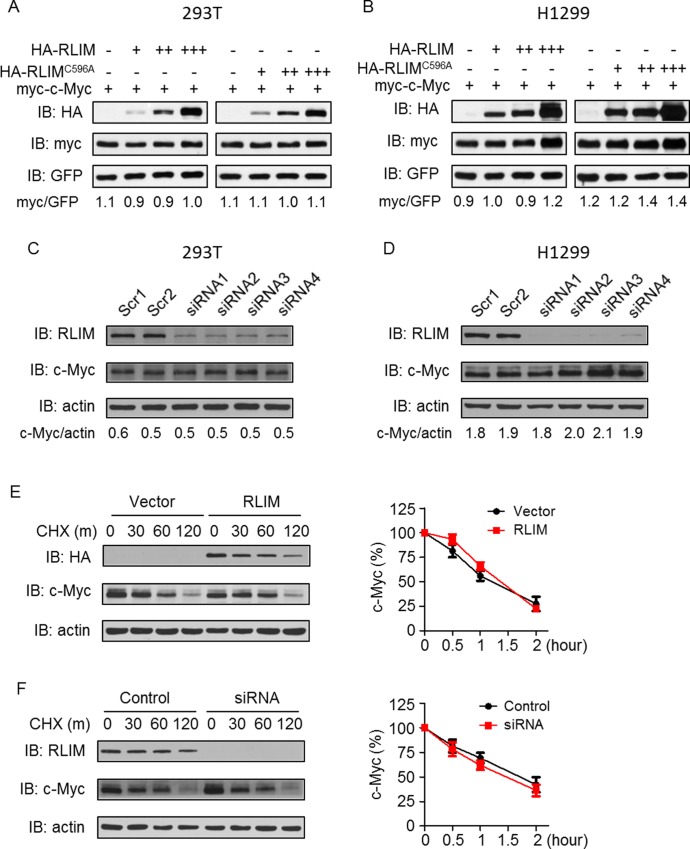
RLIM does not affect c-Myc protein degradation. (A) and (B) 293T (A) and H1299 (B) cells were transfected with myc-c-Myc and increasing amount of HA-RLIM or HA-RLIM^C596A^ plasmids as well as GFP plasmid. Ectopic c-Myc protein was detected by anti-myc antibody. GFP was used to monitor transfection efficiency. c-Myc and GFP levels were quantified using ImageJ software and the ratios of c-Myc to GFP are shown. (C) and (D) 293T (C) and H1299 (D) cells were transfected with scramble siRNAs or siRNAs against RLIM. Endogenous c-Myc protein was detected by anti-c-Myc antibody. c-Myc and actin levels were quantified using ImageJ software and the ratios of c-Myc to actin are shown. (E) and (F) 293T cells were transfected with HA-RLIM (E) plasmid or siRNA against endogenous RLIM (F). Cells were harvested at different time points after cycloheximide treatment and subjected to WB. Quantification of c-Myc protein level relative to actin are summarized from 3 independent experiments and shown in the right panels.

One consequence of ubiquitination is the proteasome mediated protein degradation. To investigate whether RLIM regulates c-Myc protein level, we transfected 293T cells with myc-c-Myc and increasing amount of WT RLIM or RLIM^C596A^ expressing plasmids. As expected, RLIM^C596A^ did not change myc-c-Myc protein level (Fig 3A, lanes 4–8). Surprisingly, overexpression of WT RLIM did not significantly change myc-c-Myc level either (Fig 3A, lanes 1–4). Similar results were also observed in H1299 cells (Fig 3B). To examine whether endogenous RLIM regulates endogenous c-Myc protein level, we designed 4 different siRNAs to knockdown endogenous RLIM. Upon transfection into 293T cells, the four siRNAs effectively reduced RLIM protein level by more than 85% (Fig 3C). However, we did not observe significant change of endogenous c-Myc protein level (Fig 3C). Same experiment in H1299 cells confirmed the observation (Fig 3D).

We then hypothesized that RLIM may regulate the turnover of c-Myc protein. To this end, we transfect 293T cells with RLIM expression plasmid or RLIM siRNAs, blocked new protein synthesis by cycloheximide (CHX) and harvested cells at different time points for WB. We found that RLIM was relatively stable with a half-life of about 60–120 minutes (Fig 3E and 3F). However, we did not observe significant changes of c-Myc turnover rate in either RLIM overexpression or knockdown conditions (Fig 3E and 3F), indicating that RLIM may not regulate c-Myc on the protein level.

### RLIM negatively regulates c-Myc function

**Fig 4:**
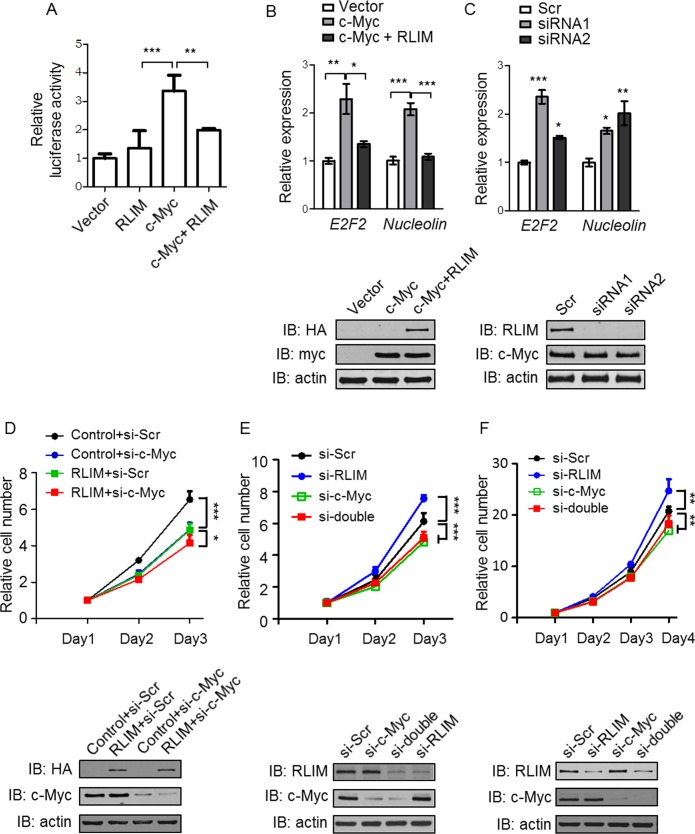
RLIM negatively regulates c-Myc transcriptional activity. (A) 293T cells were transfected with an E-box-luciferase (Firefly) reporter plasmid and a Renilla luciferase plasmid (as an internal control) together with c-Myc and RLIM plasmids as indicated. Firefly luciferase activity was measured and normalized by Renilla luciferase activity. Three independent experiments were conducted with similar results and one representative result is shown. Data is presented as mean ± SD. **p<0.01, ***p<0.001. (B) and (C) U2OS cells were transfected with c-Myc and RLIM plasmids as indicated (B) or with siRNAs against RLIM (C). Real-time PCR was carried out to examine *E2F2* and *Nucleolin* gene expression. Three independent experiments were conducted with similar results and one representative result is shown. Data is presented as mean ± SD. *p<0.05, **p<0.01, ***p<0.001. (D) RLIM overexpression and control stable U2OS cell lines were transfected with scramble or c-Myc siRNAs. 2 days after transfection, cells were plated in 96 well plates. Cell growth were monitored by cell counting kit-8. (E) U2OS cells were transfected with scramble siRNA or siRNAs against RLIM and/or c-Myc as indicated. 2 days after transfection, cells were plated in 96 well plates. Cell growth were monitored by cell counting kit-8. Three independent experiments were conducted with similar results and one representative result is shown. Data shown is relative cell number as compared to day 1 after plating. Data is presented as mean ± SD. *p<0.05, ***p<0.001. Lower WB figures show the expression of RLIM and c-Myc at day 3 after plating. (F) H1299 cells were transfected with scramble siRNA or siRNAs against RLIM and/or c-Myc as indicated. 2 days after transfection, cells were plated in 96 well plates. Cell growth were monitored by cell counting kit-8. Three independent experiments were conducted with similar results and one representative result is shown. Data shown is relative cell number as compared to day 1 after plating. Data is presented as mean ± SD. **p<0.01. Lower WB figure show the expression of RLIM and c-Myc at day 4 after plating.

Regulation of protein activity is another function of ubiquitination. To examine whether RLIM regulates c-Myc function, we firstly employed a luciferase reporter system in which the expression of Firefly *luciferase* gene is controlled by c-Myc binding element (E-box). We transfected 293T cells with the reporter and a control construct (Renilla luciferase) in combination with HA-RLIM and myc-c-Myc plasmids alone or together. c-Myc overexpression increased Firefly luciferase activity by three folds (Fig 4A). Interestingly, co-expression of RLIM completely abolished c-Myc effects on Firefly luciferase activity (Fig 4A). This suggests that RLIM negatively regulates the transcriptional activity of c-Myc. We further examined whether RLIM regulates c-Myc target gene expression. We transfected U2OS cells with c-Myc expression vector alone or together with RLIM expression construct and examined the expression of two typical c-Myc target genes by real-time PCR. Consistent with the luciferase reporter assay results, c-Myc overexpression increased *E2F2* and *Nucleolin* mRNA levels by two folds, while co-expression of RLIM completely abolished c-Myc effects (Fig 4B). To investigate the effects of RLIM on c-Myc activity in physiological conditions, we utilized two different siRNAs (as used in Fig 3C and 3D) to knockdown endogenous RLIM. Real-time PCR revealed a 1.5 to 2.5 fold increase of *E2F2* and *Nucleolin* mRNA levels upon knockdown of RLIM (Fig 4C). Collectively, these data suggest that RLIM is a negative regulator for c-Myc.

As c-Myc is known to regulate cell proliferation, we hypothesized that RLIM may control cell proliferation by regulation of c-Myc. To this end, we constructed U2OS stable cell lines that overexpress either RLIM or empty vector and monitored their proliferation. We found that overexpression of RLIM significantly reduced cell growth rate when compared with control cell line (Fig 4D, upper panel. Control+si-Scr vs. RLIM+si-Scr). Importantly, concomitant knockdown of endogenous c-Myc almost completely abolished RLIM effects on cell growth (Fig 4D, upper panel. Control+si-c-Myc vs. RLIM+si-c-Myc). WB confirmed RLIM overexpression and knockdown efficiency of c-Myc (Fig 4D, lower panel). To further confirm this observation, we knocked down c-Myc and/or RLIM by siRNAs in U2OS cells and monitored their effects on cell growth. Indeed, depletion of RLIM significantly enhanced cell growth as compared to cells transfected with scramble control siRNA (Fig 4E, upper panel. si-Scr vs. si-RLIM). More importantly, concomitant ablation of c-Myc completely eliminated the effects of RLIM depletion on cell growth (Fig 4E, upper panel. si-c-Myc vs. si-double). WB confirmed the knockdown efficiency of RLIM and c-Myc (Fig 4E, lower panel). Previously, our lab found that RLIM regulates HCT116 cell colony formation in a p53-dependent manner [13]. To determine whether p53 was involved in the present experimental settings, we utilized the p53-defecient cell line, H1299, to repeat the proliferation assay. We depleted RLIM and/or c-Myc by siRNAs and monitored cell growth. Interestingly, we got similar results as in U2OS cells (Fig 4F, upper panel). Knockdown efficiency is confirmed by WB (Fig 4F, lower panel).These results confirmed our hypothesis that RLIM restrains cell growth by negatively controlling c-Myc activity, at least in some cell lines.

## Discussion

In this study, we revealed a novel function of RLIM as an E3 ubiquitin ligase for c-Myc. We found RLIM interacts with c-Myc in cells by reciprocal immunoprecipitation. The interaction was confirmed *in vitro* by GST-pulldown assay using purified proteins indicating a direct association of the two. We further showed that RLIM can promote polyubiquitination of c-Myc in cells. Compared to previously identified E3 ligases for c-Myc, RLIM showed some novel properties. It catalyzes polyubiquitination of c-Myc, but does not lead to c-Myc degradation. Instead, RLIM attenuates c-Myc transcriptional activity towards at least some of its target genes (*E2F2* and *Nucleolin*). Moreover, RLIM restrains cell proliferation through regulation of c-Myc, as depletion of c-Myc abolished the effects of RLIM on cell growth. *MYC* is documented to be deregulated in up to 70% of human cancers [15]. Thus, our results provide evidence that RLIM could function as a tumor suppressor by controlling the activity of one of the most important oncoprotein. It will be of great importance to investigate whether RLIM is mutated or dysregulated in various type of cancers, especially in those without c-Myc dysregulations. Indeed, we found that RLIM expression is downregulated in certain percent of hepatocellular carcinoma patient samples (Pingzhao Zhang, unpublished data). This suggests that RLIM may serve as a potential drug target in treatment of certain type of cancers.

We found that expression of WT RLIM, but not catalytic dead mutant RLIM, increased c-Myc polyubiquitination in cells. However, we cannot exclude the possibility that RLIM promotes c-Myc polyubiquitination through regulation of other proteins. An *in vitro* ubiquitination assay using purified proteins will definitely help to clarify this. c-Myc exerts its transactivation function by heterodimerization with Max [34]. The heterodimer then recognizes E-box elements within target gene promoters or enhancers and recruits various transcriptional cofactors to promote gene expression [35]. We found RLIM-mediated polyubiquitination of c-Myc attenuates its transactivation activity. It will be interesting to investigate whether ubiquitination of c-Myc blocks its recruitment to target gene promoters or which step in the transactivation process is affected. RLIM was proposed to mediate ubiquitination-dependent cofactor exchange on LIM homeodomain transcription factors [36]. Probably the same mechanism is also underlying its regulation of c-Myc activity. However, further experiments need to be carried out to prove this model. c-Myc regulates various biological processes including DNA replication, RNA transcription and elongation, cell growth and proliferation, metabolism, ribosomal biogenesis, genome stability etc. [14, 15, 34, 37–47]. Here, we provided evidence that RLIM participates in cell proliferation control through regulation of c-Myc. It will be intriguing to examine whether RLIM also participates in other biological processes regulated by c-Myc.

Recently, our lab revealed an interplay between RLIM and p53. It is very interesting that RLIM interacts with two of the most important cancer-relevant pathways. Further investigation needs to be done to fully elucidate the role that RLIM plays in coordinating the most important tumor suppressor, p53 and oncoprotein, c-Myc.
